# Abdominal actinomycosis with multiple myeloma: A case report

**DOI:** 10.3892/ol.2014.2375

**Published:** 2014-07-23

**Authors:** VEHBI ERCOLAK, SEMRA PAYDAS, MELEK ERGIN, BERNA T. ATES, BERNA B. DUMAN, MERAL GUNALDI, CIGDEM U. AFSAR

**Affiliations:** 1 Department of Medical Oncology, Faculty of Medicine, Harran University, Şanlıurfa 63300, Turkey; 2 Department of Oncology, Faculty of Medicine, Çukurova University, Adana 01330, Turkey; 3 Department of Pathology, Faculty of Medicine, Çukurova University, Adana 01330, Turkey; 4 Division of Medical Oncology, Adana Numune Research and Training Hospital, Adana 01030, Turkey

**Keywords:** actinomycosis, multiple myeloma, immune-suppression

## Abstract

Actinomycosis is a chronic suppurative infection, for which immune suppression is a predisposing factor. In unusual cases, this disease may present as an abdominal wall involvement simulating a soft tissue tumor as seen in the present case. The presented patient had no signs of trauma or surgical approach and the pathology was considered to be a primary abdominal wall actinomycosis. Preoperative diagnosis is difficult due to the nonspecific nature of clinical presentation, radiographic and laboratory findings. Surgery combined with antibiotic treatment is a curative approach for this relatively rare infection. Surgeons must be aware of this disease in order to ensure correct diagnosis and to prevent performing any unnecessary procedures. The present study describes a case of abdominal actinomycosis with multiple myeloma, together with a review of important points related to this disease.

## Introduction

Actinomycosis is a chronic suppurative granulomatous infection characterized by abscess and fistula formation, and caused by aerobic or microaerophilic bacteria (1,2). *Actinomyces* is a gram-positive bacteria without a capsule and spores (3). Injury to the mucosal barrier is of critical importance to the pathogenesis of the disease (1), since this is the primary entrance for actinomycosis to invade (4). Dental procedures, surgery, endoscopic interventions and trauma may result in impairment to the mucosal barriers (5). Poor oral hygiene, immune-suppression and long-term intra-uterine devices are the predisposing conditions for this infection (6,7). Actinomycosis has been reported in patients with lymphoma, leukemia, renal failure and renal transplantation, and long-term steroid users due to immune-suppression (8,9). Primary actinomycosis of the anterior abdominal wall is uncommon. The current study presents a case of primary abdominal wall actinomycosis in a 63-year-old male with multiple myeloma, as well as a review of the literature. Patient provided written informed consent.

## Case report

**Figure 1:**
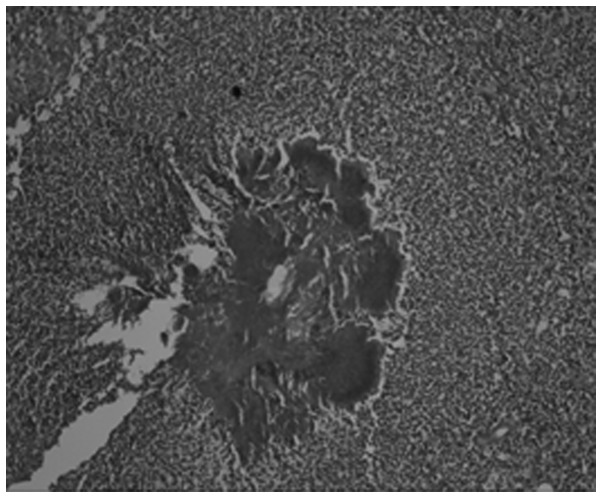
Bacteria colonies consistent with *Actinomyces*. Hematoxylin and eosin staining; original magnification, ×100.

**Figure 2:**
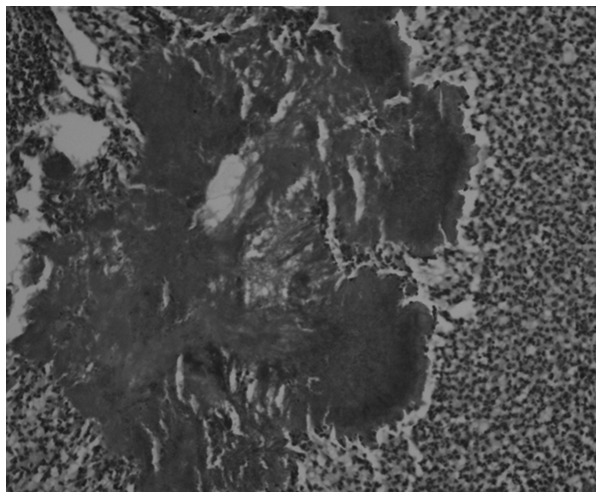
Bacteria colonies consistent with *Actinomyces*. Hematoxylin and eosin staining; original magnification, ×200.

A 63-year-old male was admitted to the Department of Medical Oncology of the Medical Faculty of Çukurova University (Adana, Turkey) with abdominal pain and weight loss (10 kg) for two months. The patient had been diagnosed with multiple myeloma and had been treated by three cycles of vincristine, doxorubicin and dexamethasone plus zoledronic acid. A suprapubic mass (3 cm diameter) was found by physical examination, and computerized tomographic scans showed a cystic mass on the rectus abdominis muscle. Repeated fine needle aspirations were non-diagnostic and a biopsy showed fibro-adipous tissue. An excision of the mass was performed under general anesthesia, and a mass biopsy was reported as an active chronic suppurative infection and actinomycosis (Figs. 1 and 2). Parenteral ampicillin was prescribed, which resulted in an improvement of the condition. Following the treatment of actinomycosis, bortezomib treatment was started for multiple myeloma.

## Discussion

*Actinomyces* are a member of oropharyngeal flora and *Actinomyces israelii* is the most frequently found microorganism (1). *A. israelii* has been identified in the female genital tract, gastrointestinal system and bronchi, and is thought to be an opportunistic organism (5,10,11). *Actinomyces* penetrate the mucosae and promote the generation of a slow-growing abscess, pseudo-tumor formation and fistulization (10). Infections progress locally rather than hematogenously (5). The most frequently observed clinical presentations are oral-cervico-facial, thoracic, abdominal and pelvic disease but disseminated and central nervous system infections may be seen (1,2,4,6,7). Primary hepatic, splenic, gastric, pancreatic, biliary tract or intestinal infections are rare, but these forms may be seen in patients with underlying immune-suppressive disorders including leukemia, auto-immune disease, alcoholism and diabetes mellitus (11,12). In unusual cases, the disease may present as an abdominal wall involvement simulating a soft tissue tumor, as seen in the presented case study. Local infection of the abdominal wall may be detected primarily or may be secondary to clinical interventions, including surgical catheter, paracentesis catheter, endoscopic interventions and long-term intrauterine devices (10,13). In the present case, there were no signs of trauma or surgical approach performed, and the patient was therefore diagnosed with a primary abdominal wall actinomycosis.

Abdominal actinomycosis initially presents with insidious symptoms, and may appear to be appendicitis, diverticulitis, intestinal perforation, trauma, intra-abdominal foreign body, inflammatory bowel disease following colon surgery and even may simulate malignant tumors. Pelvic actinomycosis may be seen in those using an intra-uterine device, which can often cause abdomino-pelvic actinomycosis (4,6,14–17). Other risk factors include an horseshoe kidney, reno-duodenal fistula and urachal remnants (18–21). There are no specific clinical symptoms. Abdominal pain, cramps, weight loss, fatigue, fever and diarrhea may be observed (13). Lymphatic dissemination is not usual due to the diameter of the bacteria or may be detected at a late stage of the disease (7). An acute abdomen occurs in the presence of fistulization (10). The duration of symptoms are variable between one month and two years (13). Generally there is a long interval between the onset of symptoms and an accurate diagnosis (20). In the present case, the interval between the onset of symptoms and an accurate diagnosis was two months due to the use of steroids and tumor suspicion due to underlying multiple myeloma.

The diagnosis of actinomycosis is based on samples or tissue biopsies taken from lesions (4). Diagnosis is difficult due to the anaerobic culture conditions that require a specific incubation from fresh samples. With the use of specialized techniques, culture positivity is <25% (13). Histopathological examination is more useful as compared to the other methods, for an accurate diagnosis; however, in the majority of the cases, as in the present case, the diagnosis is verified following surgical procedures. Fine needle aspirations are generally insufficient, as in the present case, and therefore an excisional biopsy is necessary (19,22). Actinomycotic granules may be seen in hematoxylin and eosin stained preparations (12,13,19). The presence of sulfur granules is typical for actinomycosis but not pathognomonic, and may be seen in *Nocardia*, *Streptomyces*, *Aspergillus* and some *Staphylococcus* strains (3,14,19). Masses containing abscesses and/or low-density foci may be detected by ultrasound or computed tomography scans, but these findings may be wrongly reported as a malignant tumor (13,19,23). Nonspecific inflammatory and serological markers may be elevated but are not diagnostic (24). Preoperative diagnosis is possible in <10% of the cases due to the difficulty of culture conditions, unusual clinical presentation, lack of radiological specificity and a low suspicion index for the disease (13). Omental solid masses must be considered in the differential diagnosis. These masses may be associated with primary/secondary neoplasia or inflammatory/infectious processes (chronic appendicitis, ameboma, diverticular disease and Crohn’s disease). The most frequently observed infection is tuberculosis (14). Additionally, inflammatory pseudotumors, carcinomatosis and soft tissue sarcomas must additionally be considered in the differential diagnosis (25).

The optimal therapy for actinomycosis is the wide excision of necrotic materials and long-term antibiotic treatment (26). Surgical debridement is useful both for diagnosis and treatment of this condition (4,10,14). Currently, the first choice antibiotic treatment is penicillin (4,27). Recurrence, however, is frequent in cases treated by antibiotics without surgical debridement. Preoperative antibiotics may affect the width of the surgical margins (13) and surgeons must be aware of this entity to prevent any unnecessary procedures.

Primary abdominal actinomycosis must be considered in cases presenting with abdominal masses and underlying hematologic neoplasias. An asymptomatic and/or nonspecific presentation may lead to clinical complications.
